# Myeloproliferative Diseases as Possible Risk Factor for Development of Chronic Thromboembolic Pulmonary Hypertension—A Genetic Study

**DOI:** 10.3390/ijms21093339

**Published:** 2020-05-08

**Authors:** Christina A. Eichstaedt, Jeremias Verweyen, Michael Halank, Nicola Benjamin, Christine Fischer, Eckhard Mayer, Stefan Guth, Christoph B. Wiedenroth, Benjamin Egenlauf, Satenik Harutyunova, Panagiota Xanthouli, Alberto M. Marra, Heinrike Wilkens, Ralf Ewert, Katrin Hinderhofer, Ekkehard Grünig

**Affiliations:** 1 Centre for Pulmonary Hypertension, Thoraxklinik Heidelberg gGmbH, Heidelberg University Hospital, Röntgenstr. 1, 69126 Heidelberg, Germany; Verweyen@stud.uni-heidelberg.de (J.V.); Nicola.benjamin@med.uni-heidelberg.de (N.B.); Benjamin.Egenlauf@med.uni-heidelberg.de (B.E.); Satenik.Harutyunova@med.uni-heidelberg.de (S.H.); Panagiota.Xanthouli@med.uniheidelberg.de (P.X.); albertomaria.marra@unina.it (A.M.M.); Ekkehard.Gruenig@med.uni-heidelberg.de (E.G.); 2 Translational Lung Research Centre (TLRC), German Centre for Lung Research (DZL), Im Neuenheimer Feld 156, 69120 Heidelberg, Germany; 3 Laboratory of Molecular Genetic Diagnostics, Institute of Human Genetics, Heidelberg University, Im Neuenheimer Feld 366, 69120 Heidelberg, Germany; Christine.Fischer@med.uni-heidelberg.de (C.F.); Katrin.Hinderhofer@med.uni-heidelberg.de (K.H.); 4 Department of Internal Medicine I, Carl Gustav Carus University Hospital, Technical University of Dresden, Fetscherstraße 74, 01307 Dresden, Germany; Michael.Halank@uniklinikum-dresden.de; 5 Kerckhoff Heart and Thorax Center, Department of Thoracic Surgery, Benekestr. 2–8, 61231 Bad Nauheim, Germany; S.Guth@kerckhoff-klinik.de (S.G.); c.wiedenroth@kerckhoff-klinik.de (C.B.W.); E.Mayer@kerckhoff-klinik.de (E.M.); 6 IRCCS SDN Research Institute, Via F. Crispi 8, 80121 Naples, Italy; 7 Department of Internal Medicine V—Pneumology, Allergology and Critical Care Medicine, University Hospital of Saarland, Kirrberger Str., 66424 Homburg, Saar, Germany; Heinrike.Wilkens@uks.eu; 8 Department of Internal Medicine B—Cardiology, Intensive Care, Pulmonary Medicine and Infectious Diseases, University of Greifswald, Ferdinand-Sauerbruch-Str., 17475 Greifswald, Germany; ewert@uni-greifswald.de

**Keywords:** pulmonary vascular resistance, chronic thromboembolic pulmonary hypertension, genetic predisposition, *Janus kinase 2* (*JAK2*)

## Abstract

Chronic thromboembolic pulmonary hypertension (CTEPH) is a rare disease which is often caused by recurrent emboli. These are also frequently found in patients with myeloproliferative diseases. While myeloproliferative diseases can be caused by gene defects, the genetic predisposition to CTEPH is largely unexplored. Therefore, the objective of this study was to analyse these genes and further genes involved in pulmonary hypertension in CTEPH patients. A systematic screening was conducted for pathogenic variants using a gene panel based on next generation sequencing. CTEPH was diagnosed according to current guidelines. In this study, out of 40 CTEPH patients 4 (10%) carried pathogenic variants. One patient had a nonsense variant (c.2071A>T p.Lys691*) in the *BMPR2* gene and three further patients carried the same pathogenic variant (missense variant, c.1849G>T p.Val617Phe) in the *Janus kinase 2* (*JAK2*) gene. The latter led to a myeloproliferative disease in each patient. The prevalence of this *JAK2* variant was significantly higher than expected (*p* < 0.0001). CTEPH patients may have a genetic predisposition more often than previously thought. The predisposition for myeloproliferative diseases could be an additional risk factor for CTEPH development. Thus, clinical screening for myeloproliferative diseases and genetic testing may be considered also for CTEPH patients.

## 1. Introduction

Chronic thromboembolic pulmonary hypertension (CTEPH) is a rare form of pulmonary hypertension (PH) and is characterised by organised thrombotic material and remodelled pulmonary vasculature as well as deficient angiogenesis, altered fibrinogen resolution and endothelial dysfunction [1,2]. Together these processes result in increased pulmonary vascular resistance leading to right heart failure [3]. Hypertrophy and muscularisation of the systemic bronchial vasculature and the formation of bronchial-pulmonary shunts directing the blood away from obstructed pulmonary vessels have also been described in humans and a porcine CTEPH model [4]. The exact interplay between factors in the pathogenesis still remains unclear but the process is frequently set in motion by acute or recurrent pulmonary embolism [5]. Around 4% of all patients who survive an acute pulmonary embolism will develop CTEPH [6]. While a number of risk factors for venous thromboembolism and CTEPH overlap such as non-O-blood group, phospholipid antibodies and elevated factor VIII [7], other risk factors only predispose to venous thromboembolism and not to CTEPH such as male sex and antithrombin deficiency [7,8]. Myeloproliferative disorders such as polycythaemia vera or essential thrombocythemia can also lead to thromboembolisms [9] and were identified together with CTEPH in more than ten patients so far [10,11,12,13]. In some of these patients, a pathogenic, somatic variant in the *Janus kinase 2* (*JAK2*) gene has been detected [13] and in one case CTEPH and the myeloproliferative disease was diagnosed in the same patient at the same time [11]. Moreover, differential gene expression of more than 1600 genes was detected in pulmonary artery endothelial cells from CTEPH patients in comparison to healthy controls [14].

While pathogenic variants in the *bone morphogenetic protein receptor 2* gene (*BMPR2*) and related pathway genes [15,16] have been described in patients with pulmonary arterial hypertension (PAH) only two studies reported CTEPH patients with pathogenic variants in the *BMPR2* gene [17,18]. In addition, pathogenic variants of further PAH genes such as *ACVRL1, CAV1*, *KCNK3* and *SMAD9* have been described in a single study screening 49 CTEPH patients [18]. In contrast, earlier studies could not identify any pathogenic variant in *BMPR2* including a total of 124 CTEPH patients [19,20,21,22]. In support of a genetic predisposition three descriptions of familial CTEPH exist albeit without identification of the exact genetic cause [23,24,25]. Thus, considering these reports of identified or suspected genetic predisposition the objective of this study was to systematically screen a CTEPH cohort for PAH and candidate genes predisposing to myeloproliferative disorders for pathogenic variants. This approach identified genetic predisposition for myeloproliferative disorders as a potential risk factor for CTEPH development.

## 2. Results

### 2.1. Clinical Characterisation of Patient Cohort 

Forty CTEPH patients were diagnosed at an age of 61 ± 13 years, had a mean pulmonary artery pressure of 44 ± 13 mmHg with a pulmonary artery wedge pressure of 9 ± 5 mmHg and a pulmonary vascular resistance of 7.4 ± 3.3 Wood Units (WU) (Table 1). Most patients suffered from 1-2 acute lung embolisms prior to CTEPH diagnosis. Patients were examined for myeloproliferative diseases. Three patients (7.5%) were additionally diagnosed with either polycythaemia vera, essential thrombocythemia, or primary myelofibrosis, respectively. Within the total cohort, 56% were treated by pulmonary endarterectomy (PEA), 10% underwent balloon pulmonary angioplasty (BPA) sessions, 3% had both PEA and BPA and 31% received no invasive intervention but only targeted PAH/CTEPH medication. 

### 2.2. Genetic Characterisation of Patients

Out of 40 CTEPH patients 4 (10%) carried pathogenic variants (class V), 8 further patients (20%) carried variants of uncertain significance (VUS) in 3 PAH and 7 candidate genes (Table 2) identified by next generation sequencing (NGS). One CTEPH patient had a germline nonsense variant (c.2071A>T p.Lys691*) in the *bone morphogenetic protein rector 2* (*BMPR2*) gene. Three patients carried the same gain of function missense variant, c.1849G>T p.Val617Phe, in the *Janus kinase 2* (*JAK2*) gene, which can give rise to acute and chronic myeloproliferative diseases (Table 2). The variant was most likely a somatic, thus not inherited but an acquired variant during the lifetime. So far the p.Val617Phe variant has only been identified as a somatic variant [27]. The variant was associated with polycythaemia vera in the first patient, with essential thrombocythemia in the second patient and with primary myelofibrosis in the third CTEPH patient. Two of the three patients with the *JAK2* gain-of-function variant received a PEA while the other one was not operated due to co-morbidities. The two operated variant carriers subsequently received medical therapy as CTEPH was persisting and the co-morbid patient received a double combination therapy and long-term oxygen therapy. All variant carriers suffered from a pulmonary embolism prior to CTEPH development. Clinical characteristics of non-variant and variant carriers are given in Table 3. The prevalence of the *JAK2* pathogenic variant in the general population was estimated to be 0.1% [28]. In our cohort 3 out of 40 (7.5%) unselected CTEPH patients were carriers of the pathogenic variant significantly exceeding the expected percentage of 0-1 carriers in our cohort (*p* < 0.0001). The 95% confidence interval for this variant was 1.6%–20.4%. One further patient carried a VUS in *JAK2* which has been described to have a weak gain of function effect on JAK2 activation in comparison to the wild type protein [29]. This variant is expected to be present in the germline, thus being inherited. The father of the variant carrier died due to a pulmonary embolism following an operation. 

Sanger sequencing revealed three other patients with either the thrombophilia predisposing regulatory prothrombin gene *F2* variant c.20210G>A, the known loss-of-function variant in the factor V-Leiden gene *F5* c.1691G>A p.Arg506Glu or both variants together in a heterozygous state. No increased number of thrombosis events or pulmonary embolisms were reported for these three CTEPH patients. The variants were present in 5% of our cohort (n = 2 each). No statistically significant enrichment of these gene variants in our cohort in comparison to the database genome aggregation database (gnomAD) could be identified (*F2*: *p* = 0.15, *F5*: *p* = 0.70).

The family history of three patients revealed a pulmonary embolism in a first degree relative and a deep vein thrombosis in a relative of an additional patient. None of the corresponding index patients were carriers of a pathogenic variant (class V). However, one subject with pulmonary embolism was the relative of the variant carrier with the mildly activating *JAK2* variant of uncertain significance.

### 2.3. Clinical Characterisation of CTEPH Patient With BMPR2 Nonsense Variant

Within the cohort one patient was identified with a germline *BMPR2* nonsense variant (class V) leading to a premature stop codon (Table 2). The patient was diagnosed with inoperable CTEPH at 49 years of age (Table 3). Right heart catheterisation revealed a strongly elevated mean pulmonary artery pressure of 51 mmHg, pulmonary arterial wedge pressure of 2 mmHg, cardiac output of 4.8 l/min, cardiac index of 2.2 l/min/m^2^ and an elevated pulmonary vascular resistance of 10.2 WU. Scintigraphy showed incomplete reperfusion after lung embolisms on both sides. The diagnosis of inoperable CTEPH was confirmed by an international panel of experts and the patient was included into the CHEST study [31]. Since then the patient has been treated with riociguat and improved within six months of treatment from World Health Organization (WHO) functional class III to class II, increased 6-minute walking distance by 70 m to 470 m while reducing the Borg scale from 4 to 3. In the same time frame the N-terminal pro-brain natriuretic peptide level fell from 1386 ng/l to 203 ng/l. Echocardiography revealed an improvement of systolic pulmonary arterial pressure by 5 mmHg, of the tricuspid annular plane systolic excursion by 2 mm and a reduction of the right arterial area by 3 cm^2^. The next right heart catheterisation after 2.5 years showed a drastic improvement with halved pulmonary vascular resistance and a 40% increase of cardiac index. The latest right heart catheterisation confirmed these haemodynamic improvements particularly concerning pulmonary vascular resistance (5.50 WU), cardiac output (6.9 l/min) and cardiac index (3.1 l/min/m^2^). The mean pulmonary artery pressure remained stable at 50 mmHg and pulmonary arterial wedge pressure rose to 14 mmHg. 

## 3. Discussion

To the best of our knowledge, this is the first systematic genetic assessment in CTEPH patients using a panel based on NGS including all currently known PAH genes and further genes predisposing to myeloproliferative diseases. We could identify pathogenic variants in the *BMPR2* and *JAK2* gene, respectively, in 10% of the patients. This is the third study to report a pathogenic variant in *BMPR2* in a CTEPH patient, which is usually observed in heritable PAH patients, pointing towards a possible overlap in genetic predisposition of these two precapillary forms of PH. Moreover, this study highlights the co-occurrence of a gain-of-function variant known from polycythaemia vera, essential thrombocythemia and primary myelofibrosis in three CTEPH patients as a possible co-factor for CTEPH development. The frequency of the *JAK2* variant in our cohort was highly significantly increased compared to its normal distribution in the general population. Thus, gene panel diagnostics could also be clinically and pathophysiologically relevant for the work-up of CTEPH patients.

### 3.1. Pathogenic Variants for Thrombophilia in CTEPH

Rare pathogenic variants and common polymorphisms may contribute to an increased risk of thrombus formation or non-resolution and subsequent development of CTEPH [32,33]. The *JAK2* pathogenic variant p.Val617Phe identified in this study was most likely a somatic variant originating from genetic changes which occurred in haematopoietic progenitor cells. It may act as a risk factor for CTEPH development as it can lead to increased proliferation of myeloid cells, resulting in somatic diseases such as acute myeloid leukaemia, Budd-Chiari syndrome, or the myeloproliferative diseases primary myelofibrosis, essential thrombocythemia and polycythaemia vera. In this study it was associated with polycythaemia vera, primary myelofibrosis and essential thrombocythemia in three CTEPH patients. As a somatic variant, the predisposition to myeloproliferative disorders could not be passed on to any children as we expect germline cells not to have been affected. The pathogenic variant was previously identified in one CTEPH patient with essential thrombocythemia [13] and one CTEPH patient with primary myelofibrosis [34]. Moreover, nine further CTEPH patients were reported to have polycythaemia vera albeit without genetic testing for the predisposing *JAK2* pathogenic variant [10,11,12]. However, since this pathogenic variant is present in over 95% of polycythaemia vera patients [35] there is a high probability that these patients also carried the pathogenic variant. This supports the notion that myeloproliferative diseases may be more prevalent among CTEPH patients than previously reported. In one study 1.2% of 433 CTEPH patients presented with primary myelofibrosis [36]. However, neither sequencing data nor other non-malignant myeloproliferative neoplasms were investigated in this patient cohort. Thus, further myeloproliferative diseases might have been missed and thus the prevalence of these diseases in this cohort could have been underestimated. Nevertheless, myeloproliferative disorders were taken-up as risk factors for CTEPH development in the European Society of Cardiology / European Respiratory Society guidelines from 2009 [37] but were dropped again in the latest guidelines from 2016 [38]. However, our study supports an association with CTEPH development and a possible status as an additional risk factor.

The pathogenic variant in the *JAK2* gene c.1849G>T p.Val617Phe led to an exchange of the conserved amino acid valine by phenylalanine resulting in a loss of function within the gene’s self-inhibitory domain. Subsequently, the *JAK2* gene was constantly activated and initiated the downstream signal transducers and activators of transcription pathway [35]. Hence, this pathogenic variant resulted in a gain of function of the protein leading to myeloproliferative diseases. It is important to note that probably not the *JAK2* pathogenic variant itself has a causal effect on CTEPH manifestation, but its effects on increased erythrocytosis and increased risk of thrombosis may increase the likelihood of CTEPH development. Adir and colleagues even suggested CTEPH itself could be a first manifestation of the myeloproliferative disease [39]. Alternatively, both diseases may occur independently from each other in the same patient. 

### 3.2. Pathogenic Variants in the BMPR2 and Other PAH Genes in CTEPH

There may be some overlap between CTEPH and PAH pathophysiology concerning in situ thrombosis, even though caused by different mechanisms [40], and microvascular remodelling up to the formation of (plexiform) lesions in CTEPH patients [1]. Equally, PAH patients can also present with thrombotic lesions, in particular in smaller vessels [41]. Thus, a misclassification of CTEPH and PAH may be possible. The patient described in this study carrying a pathogenic variant in *BMPR2* was included in the CHEST study [31] and therefore diagnosed to suffer from CTEPH by an international expert panel. The identified pathogenic variant was not only relevant for the patient but also for the patient’s children as it could have been passed on to the next generation. The results of our study confirm a case report of a CTEPH patient with a pathogenic variant in *BMPR2* [17] and a larger study with 49 CTEPH patients which identified *BMPR2* pathogenic variants in about 10% of patients [17,18]. Our study and these two previous publications [17,18] support the hypothesis that genetic diagnostic testing for PAH genes and myeloproliferative disorder associated genes could assist in the clinical characterisation of CTEPH patients.

## 4. Materials and Methods

### 4.1. Study Subject Characterisation

According to current guidelines [38] all patients underwent a detailed clinical work-up to establish the diagnosis of CTEPH. Since PAH can be a differential diagnosis of CTEPH, only patients with definite diagnosis have been included in the study. The assessment included right heart catheterisation, ventilation / perfusion lung scan, computed tomography angiography and pulmonary angiography. Moreover, patients received assessments of medical history, family history, physical examination, electrocardiogram, lung function test, chest x-ray, echocardiography, WHO functional class assessment [26] and laboratory parameters. Patients were treated at expert centres for pulmonary hypertension in Heidelberg and in Dresden, Germany. Operability of the patients was determined by German expert centres for pulmonary endarterectomy ( Bad Nauheim or Homburg). Further diseases which could have contributed to CTEPH were evaluated. For the diagnosis of a myeloproliferative disease laboratory parameters were measured and the gene *Janus Kinase 2* (*JAK2*) analysed for the predisposing gain-of-function variant c.1849G>T p.Val617Phe with NGS. Thrombophilia work-up included Sanger sequencing of the predisposing regulatory prothrombin gene *F2* variant c.20210G>A (rs1799963, also termed c.*97G>A in current reference sequence NM_000506.5) and the known loss-of-function variant in the factor V-Leiden gene *F5* c.1691G>A p.Arg506Glu (rs6025, also termed c.1601G>A p.Arg534Glu in current reference sequence NM_000130.5). All subjects gave their informed consent for inclusion before they participated in the study. Patients were enrolled between the years 2016 and 2019. The inclusion in the study was on average 4 ± 3 years after initial diagnosis. This study was conducted in accordance with the current version of the Declaration of Helsinki. The Ethics Committee at Heidelberg University had no objections against this study (project identification codes 065/2001, approval date: 08 August 2001 and S-426/2017, approval date: 17 October 2017). 

### 4.2. DNA Analysis

DNA was extracted from peripheral blood of CTEPH patients (Autogene, Qiagen, Hilden, Germany) using standardised procedures. Sequence variants in patients were detected using a gene panel including PAH and genes predisposing for myeloproliferative diseases (*ACVRL1, BMPR1B, BMPR2, CAV1, EIF2AK4, ENG, GDF2, JAK2, KCNA5, KCNK3, KLF2, SMAD4, SMAD9* and *TBX4*) and further candidate genes (*ACVR1, BMP2, BMPR1A, BTNL2, CREB1, CYP1B1, EPAS1, FOXO1, HGR, ID1, ID2, ID3, ID4, IL6, KLF4, KLF5, NOTCH3, SMAD1, SMAD5, SMAD6, SMAD7, SOD2, TBX2, TMEM70, TOPBP1, VCAN, VHL* and *ZFYVE16*) based on NGS, as described previously [16]. In contrast to the previous publication, additional genes were included and the sample preparation and sequencing was based on SureSelect QXT (Agilent Technologies, Santa Clara, CA, USA). 

Variants in exonic regions and exon-intron boundaries were characterised following recommendations of the Human Genome Variation Society (version 2.15.11) and the genetic variant interpretation tool of the American College of Medical Genetics [30]. Non-synonymous missense variants with a population frequency of < 1% were assessed using four in silico prediction programmes (MutationTaster, sorting intolerant from tolerant (SIFT), align Grantham variation Grantham deviation (Align-GVGD), PolyPhen2); the impact on splice sites were evaluated utilising the prediction programmes SpliceSiteFinder-like, MaxEntScan, splice site prediction by neural networks (NNSPLICE), GeneSplicer and Human Splicing Finder (Alamut Visual 2.11, interactive biosoftware, Rouen, France). The combined annotation dependent depletion (CADD) score was calculated to consider further algorithms [42] and to exclude variants with a score < 20. Benign variants and likely benign variants (class I and II) were considered polymorphisms and not followed-up. 

### 4.3. Statistics

Variants of uncertain significance (VUS, class III) and (likely) pathogenic variants (class IV and V) in PAH genes were compared to their frequency in a presumably healthy control population, i.e., individuals listed in gnomAD [43]. Clinical parameters of CTEPH patients were given in % for frequency distributions or as mean ± standard deviation. Overrepresentation of the *F2*, *F5* and *JAK2* variant in our cohort was investigated with the Fisher’s exact test implemented in BiAS version 11.4 (epsilon-Verlag GbR Hochheim, Darmstadt, Germany). A *p*-value < 0.05 was considered statistically significant. Frequency distributions of the *F2, F5* and *JAK2* variant were provided with respective 95% confidence intervals.

## 5. Conclusions

The predisposition for myeloproliferative diseases could be a further risk factor for CTEPH development and may therefore add to the work-up of CTEPH patients. In rare cases also a genetic predisposition in PAH genes could be identified providing relevant information for the treatment regimen and also family members. Thus, genetic diagnostics may be considered also for CTEPH patients to investigate a hereditary component for pulmonary hypertension and myeloproliferative disorders.

## Figures and Tables

**Table 1:** Clinical characteristics of chronic thromboembolic pulmonary hypertension (CTEPH) patients.

Parameter	Mean ± SD or %		Cohort (n) *
Age at diagnosis (years)	61	± 13	40
Women (%)	53		40
6-minute walking distance (m)	425	± 86	34
Previous history of pulmonary embolisms (%)	87		39
Family history of thrombosis or pulmonary embolisms (%)	11		36
Presence of myeloproliferative disease (%)	7.5		40
N-terminal pro-brain natriuretic peptide (ng/l)	1893	± 4186	36
**WHO functional class** [26]			36
WHO functional class II (%)	42		
WHO functional class III (%)	58		
**Treatment**			39
Pulmonary endarterectomy (%)	33		
Pulmonary endarterectomy + medication (%)	23		
Balloon pulmonary angioplasty + medication (%)	10		
Pulmonary endarterectomy + balloon pulmonary angioplasty + medication (%)	3		
Medication only (%)	31		
**Haemodynamics**			
Mean pulmonary artery pressure (mmHg)	44	± 13	36
Pulmonary artery wedge pressure (mmHg)	9	± 5	34
Pulmonary vascular resistance (Wood Units)	7.9	± 3.6	35
Cardiac output (L/min)	4.7	± 1.1	28
Cardiac index (L/min/m^2^)	2.5	± 0.5	27

* n varies for each parameter. Exact numbers are listed in this column; Abbreviations: SD: standard deviation, WHO: World Health Organization.

**Table 2:** Genetic variants class III-V in CTEPH patients identified by next generation sequencing (NGS).

Gene	RefSeq ID	Exon	c.DNA	Protein	n	Classification *	Prediction Programmes	CADD Score	gnomAD (n)
*BMPR2*	NM_001204	12	c.2071A>T	p.(Lys691*)	1	Pathogenic variant (class V)	NA (nonsense)	38.0	0
*JAK2*	NM_001322194	14	c.1849G>T *^#^*	p.(Val617Phe)	3	Pathogenic variant (class V)	gain-of-function	31.0	97
*BMPR1B*	NM_001203	8	c.556T>A	p.(Ser186Thr)	1	VUS (class III)	3/4 pathogenic	23.6	3
*BTNL2*	NM_001304561	4	c.710-4_710-8 delinsCGCTC	intronic	1	VUS (class III)	NA (intronic)	NA	0
*CYP1B1*	NM_000104	2	c.164T>G	p.(Phe55Cys)	1	VUS (class III)	2/4 pathogenic	22.1	1
*IL6*	NM_000600	3	c.263A>G	p.(Asn88Ser)	1	VUS (class III)	4/4 pathogenic	22.4	3
*JAK2*	NM_001322194	24	c.3188G>A	p.(Arg1063His)	1	VUS (Class III)	2/4 pathogenic	24.8	1272
*KCNA5*	NM_002234	1	c.213_245del	p.(Asp72_Pro82del)	1	VUS (Class III)	NA (in frame deletion)	NA	147
*NOTCH3*	NM_000435	1	c.30_35dup	p.(Arg12ArgArgArg)	1	VUS (class III)	NA (in frame duplication)	NA	0
*SMAD4*	NM_005359	5	c.565C>T	p.(Arg189Cys)	1	VUS (class III)	3/4 pathogenic	23.6	99
*SMAD6*	NM_005585	1	c.538C>G	p.(Leu189Val)	1	VUS (class III)	3/4 pathogenic	25.3	1
*TOPBP1*	NM_007027	14	c.2456A>C	p.(His819Pro)	1	VUS (class III)	1/4 pathogenic	20.9	0

*^#^* Same somatic variant identified in three unrelated patients; * Variants were characterised following guidelines from the American College of Medical Genetics and Genomics [30]; Prediction programmes used: align Grantham variation Grantham deviation (Align-GVGD), sorting intolerant from tolerant (SIFT), PolyPhen2 and MutationTaster; Abbreviations: CADD: combined annotation dependent depletion, c.DNA: coding DNA, CTEPH: chronic thromboembolic pulmonary hypertension, gnomAD: genome aggregation database with 141.456 reported sequences, n: number of CTEPH patients with the variant, NA: not applicable, RefSeq ID: reference sequence identification number, VUS: variant of uncertain significance.

**Table 3:** Clinical characteristics of non-variant and variant carriers.

Parameter	Non-Variant Carriers * Mean ± SD or %		*BMPR2* Patient	*JAK2* PV Patient	*JAK2* ET Patient	*JAK2* MF Patient	*JAK2* VUS Patient
Age at diagnosis (years)	57	± 12	49	81	65	66	51
Male:Female	0.8:1		male	male	male	female	female
6-minute walking distance (m)	432	± 91	360	NA **	411	429	414
Previous history of pulmonary embolisms (%)	85		yes	yes	Yes	yes	yes
Family history of thrombosis / pulmonary embolisms (%)	8		absent	absent	absent	absent	PE
Presence of myeloproliferative disease (%)	0		absent	yes	yes	yes	absent
N-terminal pro-brain natriuretic peptide (ng/l)	1566	± 4045	1386	12.630 ***	2975	2533	104
WHO functional class [26] II:III	0.9:1		III	III	II	II	III
PEA (%) PEA + medication (%) BPA+ medication (%) PEA + BPA + medication (%) Medication only (%)	38 18 12 3 29		riociguat	riociguat, macitentan, LTOT	PEA; riociguat, macitentan	PEA; riociguat	PEA; riociguat
Mean pulmonary artery pressure (mmHg)	45	± 14	51	45	35	32	41
Pulmonary artery wedge pressure (mmHg)	9	± 5	2	8	6	12	10
Pulmonary vascular resistance (Wood Units)	7.7	± 3.6	10.2	8.6	4.6	4.4	6.2
Cardiac output (L/min)	4.7	± 1.2	4.8	NA	5.0	4.5	5.0
Cardiac index (L/min/m^2^)	2.5	± 0.6	2.2	2.3	2.9	2.5	2.6

* n varies for each parameter; ** Patient suffered from arthrosis and had difficulty walking; In addition, the same patient had a chronic renal insufficiency; Abbreviations: *BMPR2*: *bone morphogenetic protein receptor*, BPA: balloon pulmonary angioplasty, ET: essential thrombocythemia, *JAK2*: *Janus kinase 2,* LTOT: long-term oxygen therapy, MF: myelofibrosis, NA: not available, PE: pulmonary embolism, PEA: pulmonary endarterectomy, PV: polycythaemia vera, SD: standard deviation, VUS: variant of uncertain significance, WHO: World Health Organization.

## Abbreviations

BMPR2	Bone morphogenetic protein receptor 2
BPA	Balloon pulmonary angioplasty
CADD	Combined annotation dependent depletion
CTEPH	Chronic thromboembolic pulmonary hypertension
DOAJ	Directory of open access journals
gnomAD	Genome aggregation database
JAK2	Janus kinase 2
MDPI	Multidisciplinary Digital Publishing Institute
NGS	Next generation sequencing
PAH	Pulmonary arterial hypertension
PEA	Pulmonary endarterectomy
PH	Pulmonary hypertension
VUS	Variant of uncertain significance
WHO	World Health Organization
WU	Wood units
